# The Effect of Geographical Proximity on Scientific Cooperation among Chinese Cities from 1990 to 2010

**DOI:** 10.1371/journal.pone.0111705

**Published:** 2014-11-03

**Authors:** Haitao Ma, Chuanglin Fang, Bo Pang, Guangdong Li

**Affiliations:** Institute of Geographic Sciences and Natural Resources Research, Chinese Academy of Sciences, Beijing, China; Universidad Veracruzana, Mexico

## Abstract

**Background:**

The relations between geographical proximity and spatial distance constitute a popular topic of concern. Thus, how geographical proximity affects scientific cooperation, and whether geographically proximate scientific cooperation activities in fact exhibit geographic scale features should be investigated.

**Methodology:**

Selected statistics from the ISI database on cooperatively authored papers, the authors of which resided in 60 typical cites in China, and which were published in the years 1990, 1995, 2000, 2005, and 2010, were used to establish matrices of geographic distance and cooperation levels between cities. By constructing a distance-cooperation model, the degree of scientific cooperation based on spatial distance was calculated. The relationship between geographical proximity and scientific cooperation, as well as changes in that relationship, was explored using the fitting function.

**Result:**

(1) Instead of declining, the role of geographical proximity in inter-city scientific cooperation has increased gradually but significantly with the popularization of telecommunication technologies; (2) the relationship between geographical proximity and scientific cooperation has not followed a perfect declining curve, and at certain spatial scales, the distance-decay regularity does not work; (3) the Chinese scientific cooperation network gathers around different regional center cities, showing a trend towards a regional network; within this cooperation network the amount of inter-city cooperation occurring at close range increased greatly.

**Conclusion:**

The relationship between inter-city geographical distance and scientific cooperation has been enhanced and strengthened over time.

## Introduction

In the 21st century, along with the development and popularization of new information and telecommunication technologies, the spatial scale of people’s outreach has greatly increased; conversely, the obstacles to communication offered by spatial distance have become weaker. In Friedman’s view, the world is flat, meaning that people all around the world are now able to draw increasingly closer to one another, through the use of mobile phones, the internet, and open-source programming [1]. Some scholars have even proposed the “death of geography” or the “death of distance” [2]–[4]. However, whether geographical distance has really met its “demise” is a topic of common concern in many fields.

There are two views about the relationship between geographical proximity and spatial connection. One is that geographical space no longer plays a decisive role in actors’ communication, mainly because modern telecommunication and transportation technology can overcome spatial barriers in order to instead build links [5], [6]. Another view suggests that despite globalization, geographical proximity is still a prime driving force behind actors’ interrelation activities – that is, many interactions still occur between geographically adjacent actors [7], [8]. Researchers have undertaken multiple studies on the relationships between geographical proximity and entrepreneurs [6], enterprise cooperation [9], [10], the corporate-university innovation connection [11], research institute cooperation [12], social contact [13], disease spread [14], and technology transfer [15], all of which concluded that geographical proximity did affect the formation of the relationship between these actors, albeit to varying degrees. However, in the era of the knowledge economy, surprisingly little attention has been paid to the relations between geographical proximity and scientific cooperation [16], [17].

In recent years, with the development of scientometrics, the number of studies addressing knowledge transfer, scientific cooperation, and knowledge networks, using journal article data has gradually increased [18]–[21], helping research institutes to establish scientific alliances and promote the development of science policy. In the process of building an innovation-oriented country, an increasing number of Chinese cities are making great efforts to become innovative cities, and local governments are eager to build scientific alliances in order to improve their level and enhance their influence within the national knowledge innovation network. However, few policy designs exist for promoting cooperation at the city level or considering the impact of geographical distance on the degree of cooperation, and existing geographical proximity studies often neglect to consider the important issue of geographical scales [9].

Therefore, by studying inter-city spatial distance and scientific cooperation in China, this paper attempts to answer the following questions:

1. Does geographical proximity have an effect on scientific cooperation between Chinese cities, and if so, what is its impact, and how does it change?
2. When considering the influence of geographical proximity on inter-city scientific cooperation, are there certain spatial scales at which spatial distance cooperation increases significantly?
3. What are the reasons behind the impact exerted by geographical proximity on inter-city scientific cooperation?

## Materials and Methods

### Materials

With the development of bibliometrics and the establishment of periodical databases, studies of knowledge flow using statistics of published papers have become common [20], [22], [23]. Within that existing body of knowledge, cooperatively authored papers constitute important material in exploring the exchange of knowledge, knowledge cooperation, and knowledge networks [19], [24], [25]. In this paper, the degree of inter-city scientific cooperation is reflected by the number of cooperatively authored papers being produced between cities, which are considered instances of cooperation. This is a method that is widely used in research, due to the objectivity and availability of data. Each paper contains information about the authors’ institutes or (and) working locations; as a result, the Papers Database is highly suitable for studying the relation between inter-city cooperation and geographical distance.

In this paper, Chinese cities were selected to represent scientific cooperation network nodes. Compared with other countries, China is considered a superpower with representative features in science [26]–[28], due to its rapid development of scientific fundamentals. This is reflected by the 5,203 papers authored by Chinese researchers that were published in top international academic journals in 2010, a number which places China in second place in the world in terms of publication rates in such journals. This study used data on the number of published scientific research papers in 2010 in order to select the 60 most active research centers in China (Table 1). Almost all the capital cities and municipalities were selected, because they are national and regional scientific research centers. Here, although Taiwan is an inalienable part of China, little scientific cooperation occurs between its cities and cities from the mainland due to the administrative jurisdiction; thus, cities from Taiwan were not considered. It should be noted that Urumqi and Lhasa are capital cities of Xinjiang Uygur Autonomous Region and Tibet Autonomous Region respectively, as well as two important research centers in western China, hence, in order to show the whole Chinese geographical network, these cities are also represented in the following figures which describe the Chinese inter-city scientific cooperation network. However, Urumqi and Lhasa are two isolated research centers without other case cities within 1000 km, so it is considered that these isolated centers have no possibility to establish relationships with nearby centers. As a result, in order to avoid possible errors in the results in terms of the correlations between cooperation levels and distances, Urumqi and Lhasa are excluded from the numerical analysis.

**Table 1:** The 60 selected node cities and their rank of indicators.

Name	RAP	RCU	RGDP	RPS	RRDS	Name	RAP	RCU	RGDP	RPS	RRDS
Beijing	1	1	3	1	2	Hohhot	25	28	178	83	63
Nanjing	2	13	27	11	14	Guiyang	41	25	118	70	102
Shanghai	3	4	2	2	1	Fuzhou	38	21	43	23	28
Guangzhou	5	3	6	4	3	Yinchuan	27	35	236	82	150
Wuhan	7	2	13	9	11	Xuzhou	30	58	21	40	32
Hangzhou	11	19	18	6	8	Yantai	42	41	48	58	21
Chengdu	10	13	4	12	9	Ningbo	29	33	32	14	15
Xi'an	8	8	22	7	27	Tangshan	39	52	34	220	17
Chongqing	4	7	1	13	7	Changzhou	33	45	102	21	31
Tianjin	9	6	5	8	5	Guilin	28	45	83	78	108
Changchun	6	24	31	20	36	Xining	32	45	223	204	193
Changsha	13	9	46	17	16	Daqing	40	70	174	55	40
Lanzhou	14	25	142	52	104	Zhenjiang	53	81	164	47	57
Zhengzhou	16	15	20	22	20	Haikou	56	41	233	103	204
Shenyang	15	16	28	19	19	Luoyang	37	122	53	44	46
Harbin	12	9	8	18	29	Qinhuangdao	48	70	170	180	139
Jinan	19	5	50	16	23	Jingzhou	58	45	165	76	161
Kunming	24	18	54	42	53	Lhasa	45	96	282	235	280
Nanchang	22	11	89	32	47	Lianyungang	50	122	113	86	98
Hefei	26	12	35	24	30	Wenzhou	34	70	15	38	34
Qingdao	21	25	19	15	12	Baotou	52	45	189	54	39
Dalian	17	22	51	29	18	Foshan	43	122	40	25	13
Suzhou	18	29	9	5	6	Dongguan	44	81	25	31	22
Urumqi	56	30	167	87	81	Zhongshan	57	81	166	39	62
Nanning	54	23	44	48	61	Quanzhou	55	31	26	66	24
Taiyuan	31	19	124	26	69	Jiaxing	46	81	107	28	48
Shenzhen	20	52	10	3	4	Zhuhai	47	41	251	65	100
Xiamen	23	31	147	35	52	Baoji	51	122	137	91	124
Shijiazhuang	35	16	11	27	25	Liuzhou	49	58	134	73	86
Jilin	45	52	76	112	71	Wuxi	36	38	10	55	10

Sources: All data are from 2011, the explanation of Abbreviations as follows:

RAP: Rank of number of articles published, obtained from CNKI.

RCU: Rank of number of colleges and universities, obtained from the 2012 China City Statistical Yearbook.

RGDP: Rank of GDP, obtained from the 2012 China City Statistical Yearbook.

RPS: Rank of population size, obtained from the 2012 China City Statistical Yearbook.

RRDS: Rank of number of R&D staff, obtained from the website of the National Bureau of Statistics of China’s 2011 R&D Census Report (http://www.stats.gov.cn/tjgb/rdpcgb/index.htm).

The cooperatively authored paper data came from the international periodicals database of the Web of Knowledge (http://isiknowledge.com/), which is one of a new generation of web-based academic information resource integration systems. This database includes three famous citation databases (SCI, SSCI, and A & HCI), and data for more than 8,500 of the most influential academic journals in the natural sciences, engineering, social sciences, arts and the humanities; as such, it embodies the level and internationalization of scientific cooperation, and can represent high-level international research cooperation between Chinese cities. Given these advantages, data obtained from the international periodicals database of the Web of Knowledge should be differentiated from that which could be sourced from the Chinese domestic database. It should be noted that this study addresses the amount of cooperatively authored papers between cities, which is only very slightly affected by the active population of individual researchers. Given that there is no relevant statistical data on this, standardized calculations used in this study do not reflect the active research population.

### Methods

1. Constructing the inter-city scientific cooperation matrix. Two types of cooperatively authored papers exist which reflect forms of inter-city scientific collaboration. One type results from situations where individual co-authors belong to different cities, and the exchange of knowledge among them is done across cities. The other occurs when one author works in two cities, and knowledge is exchanged through his or her own migration. From the sample survey, it was found that the probability of the latter situation occurring was only 0.6%, which can be neglected. Given that the number of cooperatively authored papers being produced between two cities can be explored via the Web of Knowledge, and that the degree of inter-city scientific cooperation can also be represented by the number of cooperatively authored papers between two cities (here considered to constitute an instance of cooperation), an inter-city scientific cooperation matrix can be constructed using data from the Web of Knowledge [29]. Here, five different matrices – covering the years 1999, 1995, 2000, 2005, and 2010– were built (Table S1).
2. Establishing the inter-city spatial distance matrix for all 58 cities. With the help of GIS to calculate the linear distance between the 58 city points, the spatial distance matrix was established. Considering the great changes seen in inter-city transportation distances in the latest 20 years, as well as the vast territory of China, this paper examines only straight-line distances. In the 58×58 matrix, the minimum distance is 18 km from Guangzhou to Foshan, and the maximum distance is 3,232 km from Haikou to Daqing.
3. Constructing a distance-cooperation computing model to calculate the total amount of inter-city scientific cooperation per unit distance interval. Taking into account that the maximum distance between the selected cities is 3,232 km and the width of Chinese territory from north to south and from east to west is approximately 3,600 km, the selected distance range was from 0 to 3,300 km. Because the average width of Chinese cites is approximately 100 km, the inter-city spatial distance was divided into 33 intervals, each one being 100 km. The statistical model of the cumulative amount of inter-city scientific cooperation at different spatial distance intervals was established as follows:
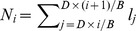

where, *N_i_* is the total number of cooperation in the *i*-th bin and i = (1,2,…,B); *D* is the maximum distance for all links between cities, and *B* is the total number of bins. *l_j_* is links between distances *D× i/B* and *D× (i+1)/B*.

## Results

### Scientific cooperation network evolution

**Figure 1:**
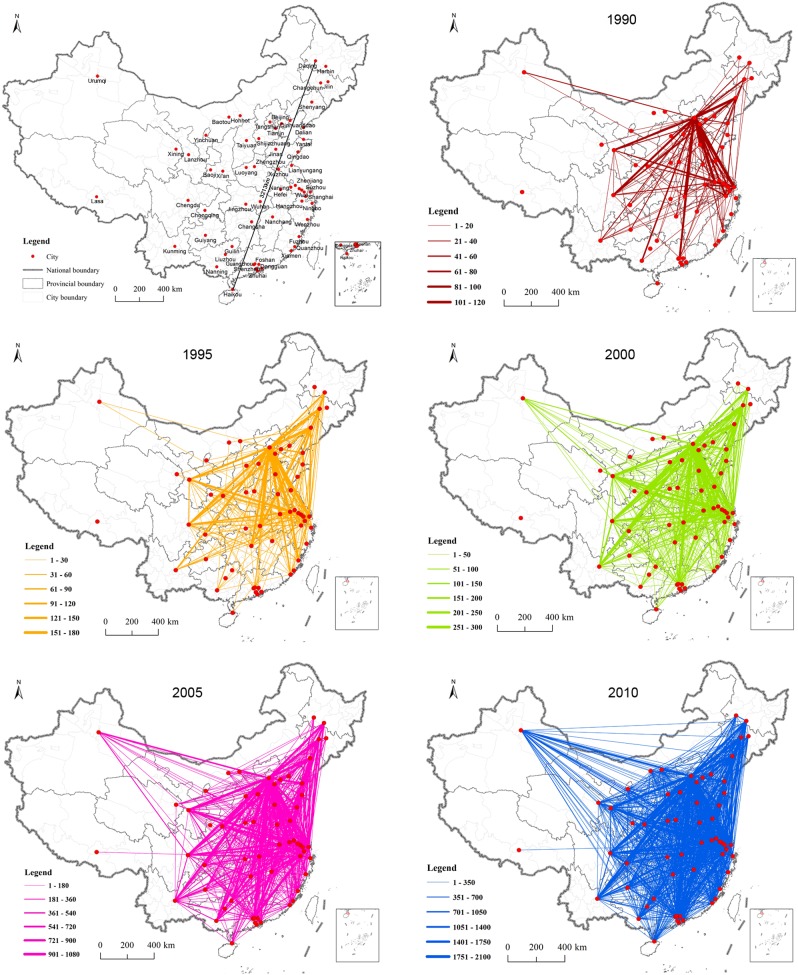
Evolution of scientific cooperation network among 60 Chinese cities from 1990 to 2010. The line between the city nodes suggests the degree of scientific cooperation.

Combined with the city’s location, 60 inter-city scientific cooperation matrices using data from 1990–2010 were used to develop a number of spatial evolution diagrams of the inter-city scientific cooperation network (Figure 1) and to explore the spatial characteristics of the network evolution. Judging from the size of the network (including the number of nodes), 46 cities made up the 1990 network, accounting for 76.67% of the total selected cities. In the 1995 network, that number increased to 54, accounting for 90% of the total selected cities; and in the 2000–2010 network, all of the selected cities were included. From these results, we can conclude that the size of the higher-level inter-city scientific cooperation network in China is expanding constantly. With respect to the level of cooperation, the average annual growth rate in inter-city cooperation was found to be 123.78%, suggesting a double growth trend. Specifically, we found 1,382 instances of inter-city cooperation to have occurred in 1990, 3,420 in 1995, 9,692 in 2000, 30,644 in 2005, and 77,558 in 2010. From the structure of the cooperation network revealed by the study, the network developed greater sophistication over time – in 1990, it maintained an obvious monocentric structure (the center was Beijing), but by 2000, it had adopted a polycentric pattern. In 2010, it had further developed towards a homogenized structure, in which it is difficult to distinguish the center of the network.

Centrality is a measure of the extent of a city in the city network, reflecting the degree of importance that a given city has in the network. By calculating the local centrality of all the cities in the Chinese scientific cooperation network over the 5 years addressed by this study (1990, 1995, 2000, 2005, and 2010), we found the overall level of the network to have grown. Further, most of the cities’ local centrality was found to have increased, especially that of super-cities like Beijing, Shanghai, and Guangzhou, indicating that most cities’ ability to carry out scientific cooperation has been enhanced during the study period. Meanwhile, the betweenness centrality results show that the capability of some super-cites like Beijing, Shanghai, and Nanjing to control knowledge decreased obviously, while such capabilities improved greatly in some regional center cities of Midwest China, like Wuhan, Chongqing, and Zhengzhou (Table 2). As a result, we can conclude that the Chinese scientific cooperation network gathers around the center, that it is developing as a regional network, and that cooperation between spatially close cities is increasing greatly.

**Table 2:** Comparison of city networks centrality in different years (Degree centrality and betweenness centrality).

City	Degree Centrality					Betweenness Centrality				
	1990	1995	2000	2005	2010	1990	1995	2000	2005	2010
Beijing	455	961	2342	7417	16480	465.70	494.83	292.34	237.08	32.35
Shanghai	199	413	1041	3356	7408	254.48	189.90	205.13	132.51	29.87
Nanjing	93	298	647	1997	4264	109.54	212.60	124.18	92.93	21.34
Tianjin	57	81	325	1195	2193	65.46	1.12	35.72	83.62	12.66
Guangzhou	31	17	305	1024	3853	51.62	2.90	126.14	36.71	56.19
Wuhan	50	156	441	1275	3230	14.54	61.42	35.35	115.31	59.43
Chongqing	3	28	96	224	1235	0.33	2.00	6.36	7.46	8.00
Zhengzhou	4	16	89	229	1033	0.00	0.00	1.98	4.88	10.25
Shijiazhuang	11	30	94	270	585	1.40	7.55	1.10	13.01	6.90
Tangshan	0	0	9	47	91	0.00	0.00	0.00	0.94	0.59
Qinhuangdao	0	6	18	77	189	0.00	0.99	0.00	4.70	3.69
Jinan	20	39	244	850	1582	10.09	10.36	12.89	45.07	8.32
Qingdao	10	6	71	573	1582	5.20	0.00	21.03	53.56	14.60
Yantai	2	9	27	147	474	0.00	52.00	10.62	7.51	2.39
Wuxi	0	16	21	126	459	0.00	13.70	4.38	7.85	11.82
Zhenjiang	2	7	8	80	398	0.75	0.00	0.00	10.03	6.27
Changzhou	1	1	10	51	258	0.00	0.00	0.00	0.12	4.61
Suzhou	20	24	100	241	863	11.07	10.91	7.63	0.64	67.64
Xuzhou	4	4	32	126	320	0.67	0.00	0.89	6.35	5.16
Lianyungang	0	0	3	24	119	0.00	0.00	0.00	0.05	1.59
Hangzhou	58	81	310	1347	2748	20.90	30.45	102.15	85.39	19.71
Ningbo	0	24	29	203	511	0.00	0.71	0.00	1.89	11.84
Jiaxing	0	1	2	28	114	0.00	0.00	0.00	0.00	0.82
Wenzhou	2	11	10	141	488	0.00	52.00	0.14	7.39	3.97
Fuzhou	13	54	87	265	903	9.25	4.24	11.11	28.51	7.44
Xiamen	4	37	62	206	814	0.60	4.97	3.22	8.57	13.28
Quanzhou	0	1	4	48	69	0.00	0.00	0.00	1.26	0.18
Shenzhen	1	1	26	258	1129	0.00	0.00	0.44	3.32	17.82
Dongguan	0	0	6	14	100	0.00	0.00	0.61	0.26	1.12
Foshan	0	0	6	28	62	0.00	0.00	0.00	0.52	0.98
Zhongshan	1	3	4	8	68	0.00	0.00	0.39	0.00	0.29
Zhuhai	0	3	2	23	50	0.00	0.00	0.00	1.61	0.47
Haikou	0	3	11	70	243	0.00	0.00	0.00	9.89	4.10
Taiyuan	6	24	87	321	784	44.00	3.29	5.66	8.63	8.78
Luoyang	1	4	16	25	340	0.00	1.21	0.66	0.00	3.13
Jingzhou	1	2	9	30	2	0.00	0.00	0.00	0.00	0.00
Changsha	9	84	160	582	1749	1.87	19.43	20.02	21.72	14.54
Nanchang	6	17	42	165	909	0.84	1.64	1.45	3.07	7.30
Hefei	30	170	505	866	2100	46.80	16.37	70.02	17.96	14.40
Harbin	12	49	127	496	1758	0.00	18.20	9.96	10.05	17.45
Daqing	2	2	7	61	202	0.00	0.00	0.00	0.27	0.43
Changchun	23	124	363	835	2015	5.77	5.11	104.07	26.81	23.81
Jilin	2	2	9	56	90	0.00	0.00	0.00	0.51	1.38
Shenyang	80	196	272	918	1948	18.10	89.59	38.84	23.63	19.35
Dalian	14	67	157	612	1505	1.46	68.73	27.28	14.31	9.16
Hohhot	1	7	10	57	329	0.00	0.83	0.33	0.37	2.33
Baotou	0	3	5	28	141	0.00	0.33	0.00	0.96	0.93
Yinchuan	3	3	8	20	133	0.00	0.00	0.29	0.00	0.43
Xi'an	13	26	307	701	2033	3.95	5.45	36.63	12.37	14.87
Baoji	1	10	7	4	23	0.00	0.00	0.00	0.00	0.10
Lanzhou	46	118	376	647	1720	8.08	22.91	33.18	14.74	12.85
Xining	1	6	18	71	274	0.00	0.00	0.00	0.10	2.08
Chengdu	40	116	339	763	2189	5.80	41.92	17.39	15.84	12.08
Guiyang	7	8	74	169	426	0.45	0.13	2.99	4.65	4.12
Nanning	15	13	29	309	646	0.00	18.27	0.00	19.71	9.89
Liuzhou	0	0	1	13	67	0.00	0.00	0.00	0.00	0.27
Guilin	2	3	35	272	444	0.00	0.00	3.18	1.65	3.16
Kunming	19	32	206	515	1245	6.30	1.99	6.93	12.83	5.49
Urumqi	7	3	41	134	570	0.00	0.00	0.34	0.92	7.03
Lasa	0	0	0	6	1	0.00	0.00	0.00	0.00	0.00

Note:Local Centrality (C*_ad_*) measures the ability of a city to carry out scientific cooperation with other cities, using the following formula:

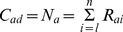
(1) where *R_ai_* is the intercity network connectivity degree between city a and city i.

Betweenness Centrality (C*_ab_*) was used to measure the controlling degree of a city on scientific knowledge. Its expression is as follows:

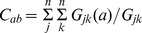
(2)

where G*_jk_* indicates the number of geodesic paths between city *j* and city *k*; G*_jk_*(*a*) describes the number of geodesic paths between city *j* and city *k*, which pass city *a*. The geodesic path is the strongest connective path between two cities.

On the whole, the inter-city scientific cooperation network in China was found to expand, to strengthen, and to become more complicated during the study period. However, given that the relationship between geographical proximity and scientific cooperation cannot be derived from the spatial features of network evolution, further study of the spatial distance and the amount of cooperation was required.

### The effect of geographical proximity

**Figure 2:**
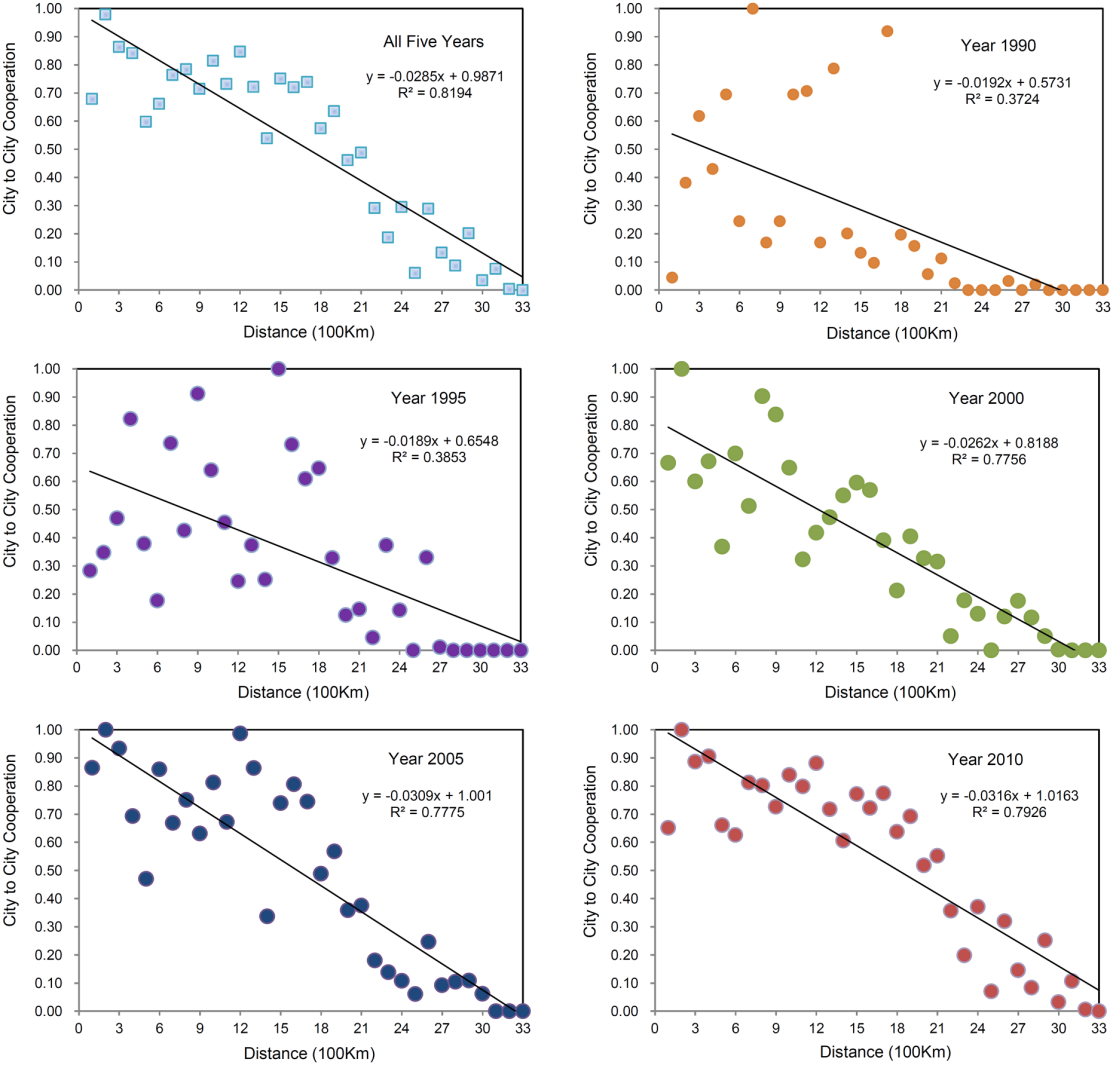
Effect of geographical proximity on city-to-city scientific cooperation in China from 1990 to 2010.

To explore the relationship between geographical proximity and scientific cooperation, and the changes that occur in that relationship, a fitting analysis of inter-city scientific cooperation at different distances was undertaken. Each fitting function was constructed using data on the amount of inter-city scientific cooperation in 33 distance intervals across the 5 years that made up the study period (1990, 1995, 2000, 2005, and 2010) (Figure 2). Initially, an analysis was made of all the years, and by accumulating the amount of inter-city scientific cooperation of five years at each distance interval, a fitting function was obtained: Y = –0.0285x+0.9871. This is a linear function with a negative slope and a relatively high fitting degree of 0.8194 (R^2^ = 0.8194). The result indicates that the distribution of city nodes was in line with the distance-decay regularity – specifically, the closer the city nodes were, the greater the inter-city scientific cooperation was, and the farther apart the city nodes were, the lesser the scientific cooperation was. In this paper, the greatest inter-city scientific cooperation occurred within a distance of 100–200 km, cumulated to 24,059; the least amount of inter-city scientific cooperation occurred within a distance of 3,200–3,300 km, cumulated to 0. Next, a fitting analysis of the distance and the amount of cooperation was undertaken to further investigate any changes in the effects of geographical proximity on inter-city cooperation. From the fitting function and its correlation coefficient (R^2^) for each year, the impact of geographical proximity on inter-city cooperation was found to gradually increase. In the years of 1990 and 1995, the correlation coefficients (R^2^) were 0.3724 and 0.3853 respectively, which indicated a weak correlation between distance and cooperation. In 2000, the correlation coefficient was 0.3724, indicating that the effect of geographical proximity had begun to manifest itself. In 2010, the correlation coefficient soared to 0.7926, suggesting a more significant influence. From the slope of each fitting function, the absolute values were 0.0192, 0.0189, 0.0262, 0.0309, and 0.0316 respectively, illustrating the way in which the effect of geographical proximity on inter-city scientific cooperation was enhanced, year-by-year.

### Spatial scales in distance decay

By examining the changing curve between the amount of scientific cooperation and inter-city distance, and the accompanying distance-cooperation distribution data, the spatial scale features of the change process can be discussed. It can be seen from the curve that the amount of inter-city scientific cooperation does not decrease continuously with increasing distance, and although the trend is in line with the distance-decay regularity, it is not a perfectly declining curve.

**Figure 3:**
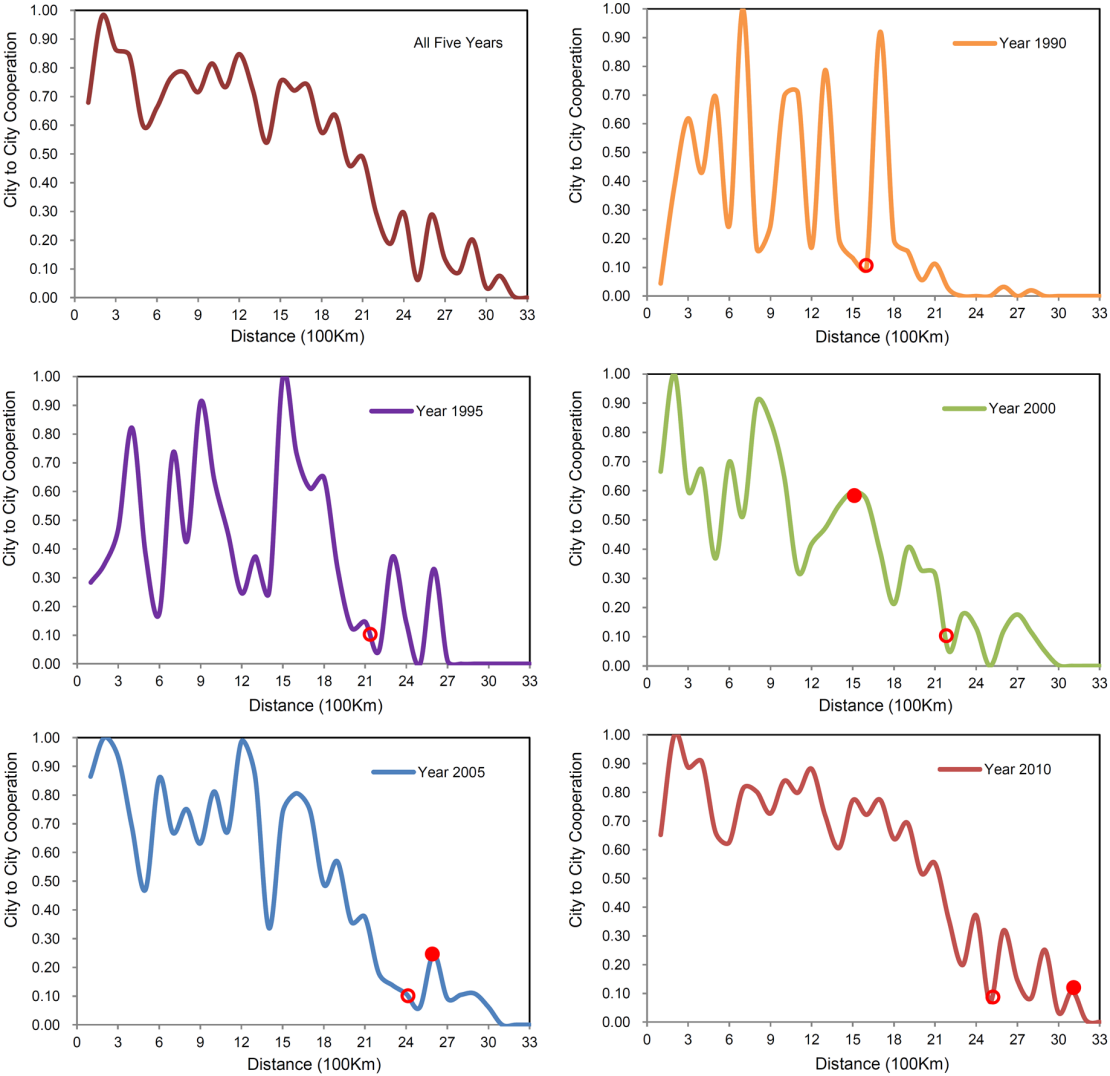
Historic evolution of spatial distance to scientific cooperation in China from 1990 to 2010. The rectangle represents different spatial levels; the red circle is the farthest point where the cooperation value that exceeds 10% of that year’s maximum value; the red dot is the point whose absolute value equals 1000.

Firstly, comparing the changing curves for the five years studied, spatial distance with a high value-point was found to move towards greater intervals. From the view of relative values, assuming that when the amount of cooperation reaches 1/10 of that year’s highest value on the curve, it is the high value. Thus, the distance corresponding to the last high value in the 1990 curve was 1,600 km, and the distance in each curve of the following 4 years was 2,100 km, 2,200 km, 2,400 km, and 2,500 km respectively, thereby demonstrating a year-by-year increase. From the view of absolute values, when the cooperation amount reaches 1,000, it is the high value. Thus, the highest value in 1990 and 1995 did not meet the standard; in 2000, the distance corresponding to the last highest value was 1,500 km; and in 2005 and 2010, the distance was 2,600 km and 3,100 km respectively – also indicating a year-by-year increase (Figure 3).

Secondly, it can be seen from the five-year cumulated change curve and the other graphs that two different distance intervals obviously existed, and within each of them, when the distance changed, the amount of inter-city cooperation changed, with significant differences. Specifically, within the distance interval of 1500 km, the amount of inter-city cooperation was found to fluctuate with increases in distance and to remain around 0.75, which indicates a weak distance decay; but outside that distance interval (i.e., beyond 1500 km), the amount of inter-city cooperation was found to decrease continuously with increases in distance, suggesting a significant distance decay. When undertaking a fitting analysis of the data in the latter distance interval, we got a linear function - Y = –0.0443X+0.7603 (R^2^ = 0.8813), showing a more obvious law of distance decay.

**Figure 4:**
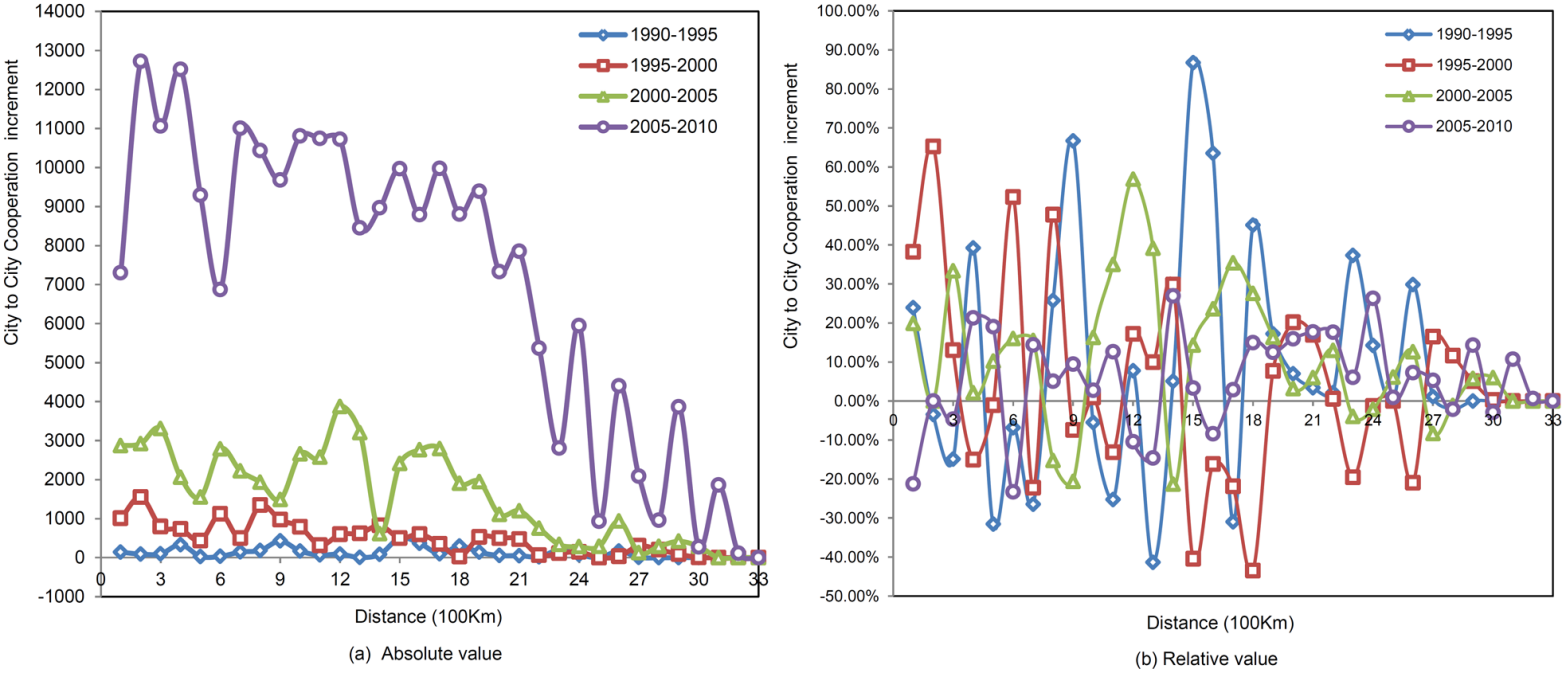
The increments of inter-city scientific cooperation within different spatial distances.

Thirdly, by comparing the increments of inter-city cooperation in the five different years studied, the spatial scale corresponding to the high increment values can be explored (Figure 4). In the period 1990–1995, the highest increment (500) appeared at 1,500 km, and an increasing trend was not obvious. During the years 1995 to 2000, the highest increment (553) occurred at 200 km, and the increasing trend was only slightly significant. From the years 2000 to 2005, the increasing trend changed greatly, and the highest increment (3,871) occurred at 1,200 km. And in the years 2005–2010, the increasing trend showed a ladder form, with the highest increment (8,965) appearing at 1,400 km. In addition, the increments fluctuated at 9,000 within the distance of 0–2000 km, which can be regarded as a high increment distance interval. Hence, it can be seen that a high increment of cooperation can occur at different distance intervals, and a significant possibility exists that newly developed inter-city scientific partnerships will develop within a distance of 2,000 km. From the relative increment, it can be inferred that the relation increment changed greatly at close range, where the average increment was also higher; further, the relation increment changed only slightly at a distance, and the average increment was also lower.

In addition, in order to compare the cooperation increment changes at different distances in different years (Table 3), the chi-square test was used to check whether significant differences existed between the two situations. Here, 1,500 km was regarded as the long-distance threshold, and the chi-square test results are shown in Table 4. Significant differences were found to exist between the long-distance and the close-distance cooperation increment in all the years. Given the actual growth witnessed in the cooperation increment, the close cooperation evidenced during 1995–2000 increased significantly faster than the long-distance cooperation, although the amount of collaboration occurring at a close range from 2000–2005 and 2005–2010 grew at a slower pace than the amount of long-distance cooperation. This suggests that the cooperation network has been developing towards a regional network structure, while obvious growth in distant cooperation appeared gradually.

**Table 3:** Comparison of cooperation increment in different years at different distances.

Year	Absolute growthvalueat close distance	Absolute growthvalueat long distance	Relative growth valueat close distance	Relative growth value at long distance
1990–1995	1623	1459		
1995–2000	12099	3429	4.7117	2.4793
2000–2005	36464	15411	3.0477	6.0907
2005–2010	150549	80757	3.9948	4.5184

**Table 4:** The chi-square test of cooperation increment change in different years at different distances.

Year	1990–1995 and 1995–2000	1995–2000 and 2000–2005	2000–2005 and 2005–2010
chi-square value	291.018^a^	1126.313^a^	169.058^a^
Sig	0.000	0.000	0.000
result	Significant difference	Significant difference	Significant difference

Note: ^a^ : 0 cells (.0%) have expected count less than 5.

## Discussion

Why does the influence of geographical proximity on scientific cooperation become stronger with the development of information technology? First, the essence of inter-city scientific cooperation lies in collaboration among researchers. For researchers’ cooperation, as social actors, another important factor of contact is social proximity [30], a measure which refers to the extent to which researchers can accept each other’s social habits, customs, and languages. An important prerequisite for social proximity is geographical proximity. Second, greater opportunities for scientific collaboration can occur among cities at a close range, and cooperation chances are more likely when two actors are in close vicinity [31]. Cities in close vicinity tend to have the same regional background, such as the same provincial government jurisdiction, urban agglomeration with close economic ties, the same ecological zone, and the same watershed, which relates these cities with more common concerns and scientific issues that need to be solved through their cooperation. Furthermore, cities in the same area tend to have a similar local knowledge pool [32], which has higher availability and will provide more opportunities for scientific cooperation. Third, inter-city internet communication and transportation infrastructures are both being developed simultaneously. According to statistics, China’s internet penetration rate has increased from 8.5% in 2005 to 45.8% in 2013, and the track traffic mileage has grown from 413 km in 2005 to 2,400 km in 2013. While internet communication infrastructure can be of great help in promoting scientific cooperation at different distances, transportation infrastructure benefits face-to-face scientific cooperation at close range. Therefore, the development of information technology not only provides greater chances for researchers to collaborate with their counterparts at a distance, but it also enhances inter-city scientific cooperation in the close vicinity. Knowledge spillovers, knowledge transfer, and scientific cooperation are more likely to occur between nearby cites [33].

Why does a significant difference exist between the changes seen in distant and in nearby inter-city cooperation? Why do spatial scales, or phases, exist in the distance decay of scientific cooperation? First, from the perspective of economics, knowledge transfer must have a geographic upper bound, largely because the marginal costs of knowledge transmissions increase with distance [34]. Hence, differences would exist between cooperation within certain geographical boundaries, and cooperation with the areas beyond those boundaries. Second, from the perspective of cultural geography, three types of knowledge diffusion exist, namely: contagious diffusion, hierarchical diffusion, and relocation diffusion, among which hierarchical diffusion should be the key factor in forming such scale features [35]. It is easier to shape a hierarchy for countries with large land area, such as China. Third, from the sociological point of view, when choosing collaborators in the same spatial scale, researchers are inclined to consider non-spatial distance factors, such as complementarity due to homogeneity issues or competitive factors [36], because of the same regional background, social environment, and local knowledge atmosphere. Fourth, transport would be an important factor affecting scientific researchers in their decisions about how far they are prepared to travel to carry out face-to-face communication. Two major options are high-speed railways and air travel; the former mode of transport is the better choice in this case, due to its convenience and price. If the longest tolerable travel time is six hours and the average speed of a high-speed railway is 250 km/h, then the distance between two cites that can be accessed by high-speed railway is 1500 km – this probably explains why 1500 km was found to be the distance split point.

## Supporting Information

Table S1 **The standardized number of cooperated papers between 60 Chinese cities.** A) in 1990. B) in 1995. C) in 2000. D) in 2005. E) in 2005.
(XLSX)

Data S1 (XLSX)
